# Treating Traumatized Offenders and Veterans by Means of Narrative Exposure Therapy

**DOI:** 10.3389/fpsyt.2015.00080

**Published:** 2015-06-22

**Authors:** Tobias Hecker, Katharin Hermenau, Anselm Crombach, Thomas Elbert

**Affiliations:** ^1^Department of Psychology, Division of Psychopathology and Clinical Intervention, University of Zurich, Zurich, Switzerland; ^2^Vivo International (www.vivo.org); ^3^Department of Psychology, Division of Clinical Neuropsychology, University of Konstanz, Konstanz, Germany

**Keywords:** trauma, violent offenders, veterans, narrative exposure, PTSD, aggression

## Abstract

Violent offenders and soldiers are at high risk of developing appetitive aggression and trauma-related disorders, which reduce successful integration into societies. Narrative exposure therapy (NET) for forensic offender rehabilitation (FORNET) aims at reducing symptoms of traumatic stress (e.g., posttraumatic stress disorder) and controlling readiness for aggressive behavior. It follows the logic of the evidence-based trauma-focused NET with special emphasis on violent acts in past and future behavior. In NET, the therapist guides the client by means of exposure through his traumatic experiences in chronological order linking the negative emotions, such as fear, shame, and disgust, to the past context and integrating the traumatic experiences into the autobiographical memory. During FORNET, we also encourage verbalization of any positive emotions and experiences linked to past violent and aggressive behaviors. This recall of positive emotions (linked to the *there* and *then*) is contrasted with feelings that emerge during the narration process (*here* and *now*). In this way, the therapist helps the client to anchor the whole range of sensory and bodily experiences, cognitions, and emotions to the contextual cues. Over the process of the therapy, we support the client to begin the role change from a violent offender to a citizen, who is capable of living a non-violent and socially adjusted life. Finally, the client develops visions and wishes for the future to support a successful integration into society. Several studies with veterans and violent youths have proven the feasibility of FORNET, found evidence of a positive outcome (recovered mental health, fewer offenses committed, less drug intake, and improved integration into civil society), and highlighted the importance of addressing the whole range of experiences while treating violent offenders or veterans.

## Introduction

Understanding the psychology of offenders may require detaching oneself from the victim’s perspective. Anecdotal evidence suggests that the perception of violence may differ remarkably between the perpetrator and victim (1): violent offenders, such as hooligans or gang members, as well as soldiers and veterans describe that under certain circumstances they have perceived the perpetration of violent acts as thrilling, fascinating, and arousing with a positive valence. Balint (2) defines this violence-related thrill as a mixture of pleasure and anxiety, with a strong confidence to master the potential danger. In his book about being a hooligan Danny Brown describes the feeling as follows:

Well, the passion to fight for your club combined with the adrenalin flush was just unbelievable. […] The violence was like heroin and it became like an addiction. It is unfair that Football-Hooligans are jailed in prison but not send into a rehabilitation clinic, where they try to help you. (3)

The literature on war and conflict reports similar experiences of soldiers anecdotally. For example, some veterans from the US who served in Vietnam described that the thirst for further combat resembled an addiction:

Combat addiction […] is caused when […] the body releases a large amount of adrenaline into your system and you get what is referred to as a “combat high”. This combat high is like getting an injection of morphine – you float around, laughing, joking, having a great time, totally oblivious to the dangers around you. […] Problems arise when you begin to want another fix of combat, and another, and another, and, before you know it you are hooked. As with heroine or cocaine addiction, combat addiction surely will get you killed. And like any addict you will get desperate and will do anything to get your fix [(4), p. 243].

Similarly, former soldiers and combatants often report that their experience of war and violence brought about a gradual transformation in their perception of violence: at first, it was frightening, but with repeated experience it became not only normal and acceptable but also even exciting and arousing (5). We have collected a number of reports of former combatants in a very recent armed conflict in the Democratic Republic of the Congo (DRC) confirming how fascinating, thrilling, and addiction-like the perpetration of violence may become:

We were sitting together, my uncle and me. We were talking about our glorious fights and then the need for fighting, the urge came up in us. It could be even at 7 o’clock at night, when it was already dark, that we took the guns and went to kill. I wanted people to know that I am a man! It is fun to plan the fight and once the enemy is defeated you feel at ease! (6)

On the other hand, studies on veterans have revealed a high posttraumatic stress disorder (PTSD) prevalence in former soldiers (7). For example, the National Vietnam Veteran Readjustment Study has shown an increased PTSD rate in veterans emphasizing that exposure to deployment-related traumatic experiences contributed strongly to the development of PTSD (8). Particularly, Vietnam veterans who reported that they had killed during deployment showed higher PTSD scores than those who did not (9). Even though attempts were made to define selection criteria to sort out those who are vulnerable for traumatization (10), trauma spectrum disorders are still common in soldiers who have fought in current armed conflicts like in Iraq or Afghanistan (11). Likewise, numerous studies focusing on the psychopathology and biography of offenders have shown that delinquents have an increased prevalence for psychological disorders compared to the general population (12) and are more frequently exposed to traumatic life events. Accordingly, studies have repeatedly reported an increased prevalence for PTSD in delinquents when compared with the general population (13). Lifetime prevalence varies between 33 and 36%, and point prevalence between 17 and 21%. In a sample of male prisoners in Switzerland, the prevalence of current PTSD was with 27% comparable to other international studies (14). Seventy-five percent of the subjects had experienced at least one event that matched the criteria for a traumatic event according to the DSM-IV but only a minority of violent offenders reported their own offenses as the most traumatic experience (15).

Treatment response to trauma-focused therapy has, however, often been poor for violent offenders and veterans (16). Feeny et al. (17) call for caution in the delivery of exposure-based PTSD treatments to violent offenders, based on clinical judgment, but limited research evidence. Kubany (18) addresses the prominence of guilt in combat-related PTSD and argues that specific attention to the combat-related guilt is required to reduce the posttraumatic stress symptoms. Thus, a special focus on offender issues might be necessary to reduce PTSD symptoms in this group. At the same time, treatment approaches may aim at controlling aggressive behavior and facilitate the role change from a potential offender to a citizen, living a non-violent and socially adjusted life. While working with violent offenders, soldiers, and veterans, it seems therefore crucial to overcome the dichotomy of victim and perpetrator in order to address the complexity of the persons’ feelings and experiences (19).

### The cycle of violence

War-affected regions as well as collective violence worldwide are characterized by escalating violence (20). In addition to the specific initiating conditions that lead to an outbreak of mass violence (in terrorism, gang warfare, war, and genocide), extreme forms of violence, including inconceivable cruelty and inhuman punitive methods shape the perpetrators’ behavior throughout cultures and regions (21). The literature distinguishes consistently between two major forms of aggression: reactive and instrumental aggression (22, 23). Reactive aggression is also known as affective, impulsive, or hostile aggression. It can be conceived as driven by anger, and occurring as a reaction to some perceived provocation or threat. If an acute threat is posed, either to oneself, one’s own children, members of one’s own community, or to one’s own resources, the disposition to harm others increases spontaneously. Instrumental aggression, however, is planned, purposeful and target- or goal-oriented.

We argue that valence is appropriate to display the differences between distinct forms of aggression. Reactive-aggressive individuals seem to reach an aversive and high-arousal state easily as they are more likely to perceive provocations as hostile or intentional (24). Reactive aggression is positively related to social exclusion and victimhood (25), which suggests an aversive and high-arousal state that could prompt aggressive behavior. In addition to reactive aggression, appetitive aggression as a subtype of instrumental aggression was introduced into the literature in an attempt of understanding the mechanisms causing extreme violence (26). Appetitive aggression was defined as the perpetration of violence or the infliction of harm upon a victim, with the aim of experiencing violence-related enjoyment through exposure to violent cues, such as the struggling of the victim. In other words, appetitive aggression can be defined as perceiving aggressive behavior toward others as fascinating, arousing, and thrilling even without gaining any external benefit (27); motivated by a direct increase in positive arousal caused by the aggressive act itself (26).

Aggressive behavior, as one facet of human behavior, aims either to dissolve an aversive state or to reach an appetitive state. Moran et al. (28) have validated this distinction of reward-driven appetitive aggression from reactive aggression in ordinary populations at the level of functional neural brain circuitry.

Approach behavior, be it for sex, drugs, or appetitive aggression is regulated in all cultures through specific societal rules and moral norms, while avoidance of threat by means of reactive aggression is generally accepted. The inhibition that prevents aggressive behavior must therefore be overcome, usually by constructing some kind of threat by a (presumed) enemy. Hence, actual aggressive behavior is frequently a combination of both forms, whereby, when an attack is possible, reactive is turned into appetitive forms of violence.

### Exposure to violence and trauma-related disorders

**Figure 1:**
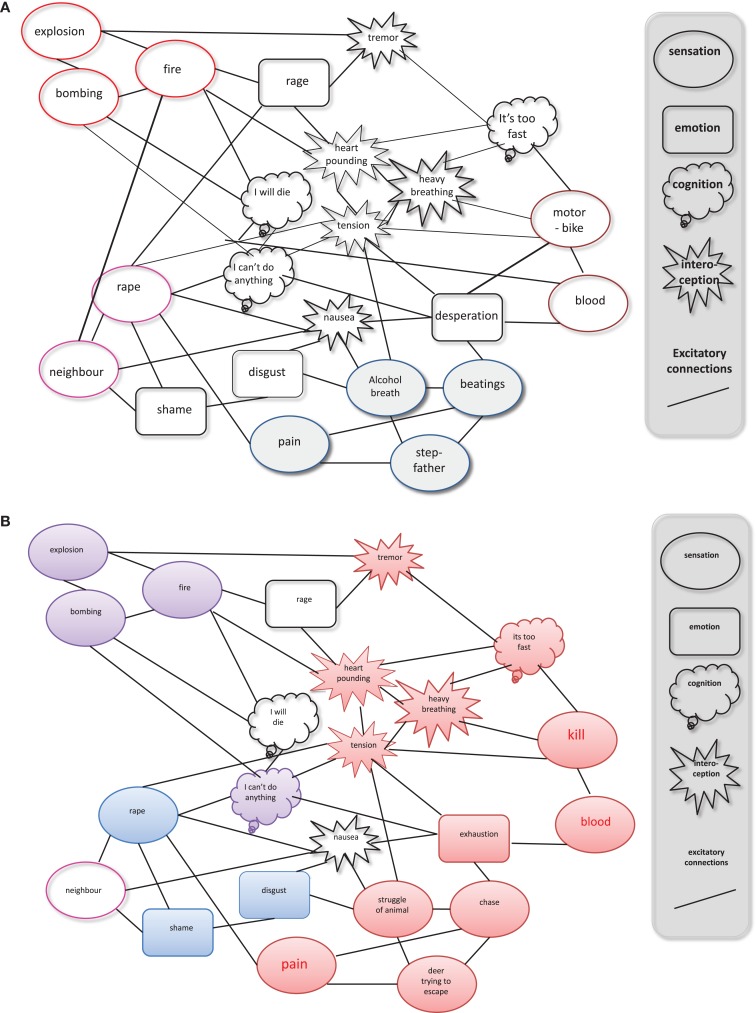
**Competition between the (A) fear network and (B) the hunting network: (A)** repeated experience of traumatic stress forms a fear network of related sensory, cognitive, emotional, and physiological memories that are detached from contextual cues such as time and location of the danger. This network is held to explain the development of most of the symptoms from the spectrum of trauma-related disorders. Thus, one of the main consequences of untreated traumatic experiences is that the emotional-sensory (“hot”) past continuously pushes into the present. The memories remain largely implicit, making it impossible for the traumatized person to talk about them, a phenomenon, which has been called “speechless terror” (34, 35). **(B)** Hunting experiences also form a network of related sensory, cognitive, emotional, and physiological memories, which may be very similar to the contents of the fear network. However, the affective valence of much of the emotional experiences and memories is exactly opposite: the fear network links the memories of the event only to negative affect, while the valence of the disposition to hunt is intrinsically positive [from Elbert et al. (26)].

Prior research has consistently shown that the greater the cumulative exposure to traumatic experiences the greater the risk of trauma-related disorders, including PTSD, depression, or substance abuse (29, 30). The brain adapts to frequent stressors and danger, such as those posed by a violent environment, by prioritizing a stress–responsive pathway. This pathway helps the individual not only to react to danger with aggression or flight but it is also related to a higher risk of mental illness (31). As exposure to different types of traumatic stressors increases, the prevalence of PTSD and other manifestations of mental illness increase. This “building-block-effect” of cumulative trauma has been found throughout many crisis regions (32, 33). Previous research indicates that life-threatening situations create an associative memory, a fear network that links highly arousing emotional-sensory and somatic memories of the traumatic experience such that a cue increases the likelihood to reactivate any recall of earlier alarm responses. With each additional traumatizing experience, the survivor increasingly perceives threats to life and integrity as being omnipresent. While the connections between reminders for traumatic stress are strengthened, the context enveloping each cue slowly disappears (Figure 1A): the “*when* and *where*” of each experience is not integrated into the network, its elements are no longer perceived as memories from a different time and place, the individual is left to experience the threat without understanding from where it is coming: this is the gateway to PTSD symptoms.

### Exposure to violence and aggressive behavior

Curtis (36) first expressed concern that abused or neglected children may become perpetrators of violence in adolescence or adulthood. While the validity of this concept cannot be assessed by direct experimental manipulation, substantial and converging evidence confirms that experiencing violence is related to expressing violence; for instance, parents who were abused as children are more likely to abuse their own children. Rates of abuse is double for parents who themselves grew up in violent environments compared to parents who did not (31). Prospective and retrospective studies on children who were abused or neglected disclosed a high incidence of later delinquency. For example, children clinically referred to residential treatment with a history of abuse scored significantly higher on measures of aggression than non-abused control children (37). Violence exposure occurring specifically in the home or community has been associated with the development of conduct problems in children. Likewise, being a victim of violence was found to be the single best predictor of juvenile violent behaviors for children in a sample of adolescents (38, 39). Finally, a large proportion of homicide offenders come from unfavorable home environments and up to 80% of subjects within delinquent samples reported witnessing violence in their childhood or adolescence.

Thus, effects of exposure to violence are exerted from early childhood onward, when plasticity for brain and mind is greatest. Developmental studies indicate that abuse and neglect are related to aggressive behavior in children from infancy onward (40). Although relatively short-term, these developmental studies provided serious ground for concern, given the literature that suggested that aggressiveness is a fairly stable personality trait and that early aggressiveness was predictive of later antisocial behavior (40). Hence violent childhood experiences may leave their mark on the brain and mind of the affected individuals, a vulnerability that interacts with future stressful experiences. Extreme or continuous stress may drive the individual into an increasingly maladaptive state with the potential for mental disorders (31). In the past few years, research has provided substantial evidence that trauma-related mental disorders are associated with reactive aggression (41, 42). Studies addressing consequences of traumatic experiences found increased impulsive aggression toward intimate partners and own children (43). Family violence is associated with a number of emotional and behavioral problems in childhood, adolescence, and adulthood (44, 45). Genetic and epigenetic studies suggested the impact of gene–environment interactions (46). For example, MOA polymorphisms seem to be related to childhood adversities and aggression (47, 48). Thus, previous research provides evidence of a cycle of violence perpetuating itself through families, and communities in interactive and complex patterns. Violent environments promote not only reactive but also future appetitively driven violent behavior.

### Adapting to perpetrating violence

Usually, control mechanisms in the frontal lobe are thought to inhibit intraspecific violence (49). Some of these inhibition mechanisms are learnt during moral development and may depend on the moral standards in a given society or culture. In gangs or militia groups, the dehumanization of the enemy and initiation rites can, however, break these moral standards (50, 51). Once the inhibition has been overcome, committing violence can become appealing fascinating and exciting (26, 52). The origins of this desire for aggression, characterized by a fascination with and an enjoyment of cruelty may lie in the development of hunting behavior (52). Human hunting behavior has evolved as a profitable strategy, and perpetrating violence against one’s own species has brought manifold evolutionary advantages (53). Nell (54) suggested an affectively positive, dopamine mediated and therefore rewarding perception of violence, is responsible for the enjoyment of violent behaviors. In line with this idea, Weierstall and Elbert (21) replicated several studies describing rewarding feelings related to the perpetration of violence in a large sample of more than 1600 former combatants and child soldiers from different conflict zones in the world. In a number of studies, appetitive aggression was associated with perpetrating violent acts (27, 52), and adapting well to violent environments (55). “*Mutilating another person*” and “*attacking a village/settlement*” emerged as items with the greatest impact for enhanced levels of appetitive aggression, the intrinsically motivated form of violence that accounts for reports of *combat high* (56). When civil socialization is replaced by socialization in a violent environment early in life, self-regulation of appetitive aggression may become deficient, leading to a higher propensity toward cruelty (57). As appetitive aggression has shown to be a major risk factor for future violent behavior (58), it may also hinder successful rehabilitation of violent offenders (59). Thus, appetitive aggression, guilt, and shame need to be addressed before ex-combatants may begin the reintegration process into society.

### Perpetration of violence and trauma-related disorders

Perpetrating violence may have a direct impact on the mental health of offenders. In a number of studies with veterans and in historical cases, MacNair (60) explored the impact of perpetrating violence on the mental health of offenders. In a study with Vietnam veterans, she found that those veterans who reported that they have killed showed higher PTSD scores than those who did not. The effect size of the group difference was large and remained significant after controlling for battle intensity (9). She concluded that perpetrating violence, particularly killing, leads to enhanced risk for PTSD. Consistent with this, in historical studies of war, Grossman (4) showed that there seems to be a great resistance in human beings to kill. Even professional torturers reported trauma-associated symptoms (61). Thus, in a number of recent studies, researchers rated perpetrating violent acts *per se* as traumatic experiences (62, 63). Following DSM-IV (64), a life event is classified as traumatic if it produces feelings of helplessness, horror, or massive fear.

However, perpetrating violence does not necessarily result in a fearful or horrified response (65). Recent studies with former combatants and child soldiers reported only low rates of trauma-related suffering (62, 66). Concurrently, the literature from several conflicts provides anecdotal evidence that under certain circumstances normal people can become extremely violent and seem not to suffer under their atrocities but rather enjoy their cruelty (4). Thus, in male offenders, exposure to and exertion of violence may not necessarily increase the likelihood of trauma-related disorders.

### Traumatic stress and appetitive aggression

Elbert and colleagues (26) suggested that, in analogy to the fear network (31, 34), perpetrators form a “*hunting network*,” linking cues related to attack and marked by approach of, rather than avoidance of violent cues. Whereas exposure to violent acts leads to an extension of the fear network, arousing or appetitive elements that arise during the perpetration of violence are integrated into the hunting network (Figure 1). The massive exposure to violence as a victim leads to an extension of a fear network, which can be triggered by re-exposure to a violent cue. This in turn then evokes a massive alarm response. By contrast, exposure to the same violent cues from the perpetrator’s perspective would form connections that are integrated with the appetitive elements of the hunting network. Thus, perpetrators may perceive violent cues as appetitive instead of aversive. This “*hunting network*” seems to stimulate appetitive arousal when a sufficient number of its memory elements have been activated by respective exteroceptive and interoceptive stimuli. However, as violence cues share many sensations, cognitions, and physiological responses with those that may also form part of the fear network, the exposure to violence can also cause severe distress to violent offenders, if the integration into the hunting network fails and memories are integrated into the fear network (Figure 1).

According to Elbert et al. (26) becoming a perpetrator could result in appetitive behavior disconnecting many of the cues, like, for instance, “blood” from the neural fear network. Instead, they become associated with the fascination for violence. This pruning of the fear network may result in a decreased vulnerability for PTSD. Due to the fact that the appetitive, fascinating element of violence seems to prevent the incorporation of the cruel, genuinely traumatizing experiences into the fear network, appetitive aggressive individuals may have a higher chance of survival in the bush (26). This idea may also explain the initially surprising findings that many violent offenders did not fall ill within the trauma spectrum (PTSD, depression, substance abuse) although they went through tremendously distressing experiences and ongoing threats of torture and death. Thus, the possibility that appetitive aggression, including planning, perception, and experience of violent acts, may promote resilience for PTSD can be explained on the basis of the competition between the networks representing the generalized fear and hunting experiences.

Consistently with this, a number of studies have found a negative relation between PTSD symptom severity and appetitive aggression. For example, in a study with genocide perpetrators in Rwanda, appetitive aggression was negatively related to PTSD symptom severity (52). The results indicated that appetitive aggression might indeed reduce the vulnerability of violent offenders for trauma-related disorders and prevent them from getting traumatized by their own atrocities. These findings were replicated in several studies with violent offenders and veterans, e.g., with child soldiers (67), demobilized militias (68, 69), World War II veterans (70), and violent youth (58). Concordantly, Elbert et al. (26) stated that appetitive aggression may buffer the risk of PTSD, as the integration of violent cues into the hunting network (and not into the fear network) may reduce the likelihood of a trigger-related activation of the fear network. On the other hand, they argue that this protective effect may wane if the offender exceeds a certain level of traumatization due to an overlap of the hunting and the fear networks. With a greater number of items linked to the fear network, comes a higher likelihood of the fear network being triggered. Consequently, the offender may experience trauma-related symptoms. Although perceiving the perpetration of violence as fascinating and arousing can lead to a substantial risk-reduction, cumulative trauma exposure will eventually trigger a trauma-related disorder (68, 69).

In conclusion, the success of offender rehabilitation can be hindered by trauma-related problems and aggressive behavior alike (71). PTSD symptoms like concentration problems, flashbacks, sleeping problems, and hyperarousal can lead to impaired functionality and a greater risk of dropping out of rehabilitation programs (72). Violent offenders who have been traumatized by their own offense present with avoidant behavior that may endanger the success of offender rehabilitation programs (15). Furthermore, trauma-related mental disorders and attraction to violence are associated with aggressive behavior. The traumatized perpetrators who are at the same time highly appetitive aggressive have an elevated risk of engaging in violent acts: impulsive violent reactions to perceived threats might then trigger the hunting network causing the perpetration of severe atrocities. Such behavior leads to interpersonal problems and can cause discontinuation of rehabilitation programs (73). However, if offenders drop out of rehabilitation programs they are at high risk of perpetrating further violent and delinquent behaviors. Therefore, we present a new approach for treating traumatized offenders and veterans that aims to overcome the dichotomy of victim and perpetrator in order to address the complexity of the persons’ feelings and experiences.

## Narrative Exposure Therapy for Forensic Offenders Rehabilitation

### Narrative exposure therapy

Narrative exposure therapy (NET) is an evidence-based short-term culturally universal intervention for trauma victims and has proven to be successful in different settings (74–76). In essence, during NET, the therapist helps the client to construct a chronological narrative of his/her whole life focusing on exposure of traumatic experiences. Hot memory (sensations, feelings, thoughts, and bodily sensations) becomes connected to the corresponding sequences in the autobiography by putting all memories into words and placing them into the narration of the event. NET focuses on the exposure of the most traumatic events. Clients who were actively involved in perpetrating violent acts also profited from NET treatment: in a study with child soldiers in Northern Uganda, NET proved superior to waiting list and an academic catch-up intervention in reducing PTSD symptoms (74). Perpetrated violence can thus be addressed if the violent act is perceived as traumatic by the perpetrator. Child soldiers are often forced to act violently, which may be perceived as traumatic. However, combatants who volunteered to join militia groups often did not perceive their own involvement in violent acts as traumatic (65). With these clients, the perpetration of violence is not explicitly addressed in NET. Consequently, veterans and former child soldiers are treated as victims of violence, neglecting that they also report positive feelings during the perpetration of violent acts (5, 26). A study comparing responders and non-responders to NET treatment in a sample of refugees in Norway showed that male refugees who reported that they had perpetrated violent acts were less likely to respond to NET treatment (16). Thus, a special focus on offender issues is necessary to reduce posttraumatic symptoms in veterans and violent offenders. For successful rehabilitation and recovery, it is, therefore, crucial to address the complexity of all feelings and experiences in therapy (19). Consequently, we have developed an adapted version of NET that addresses both traumatic experiences and perpetrated violent acts.

### Rationale of FORNET

**Figure 2:**
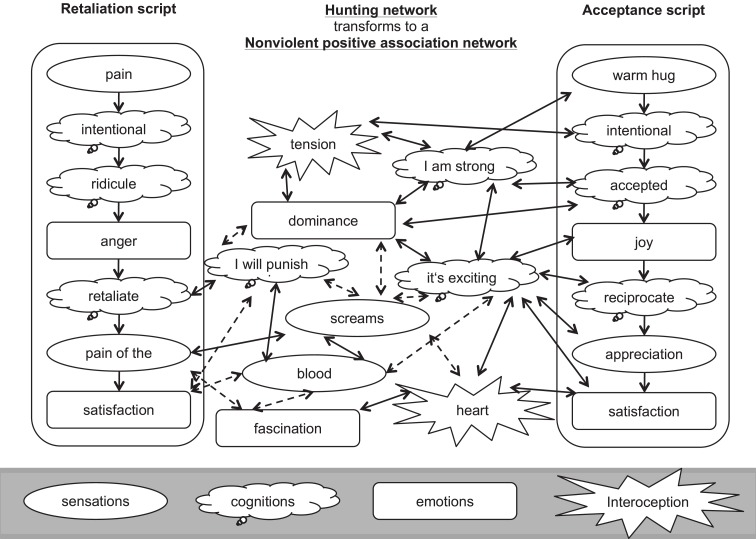
**Schematic representation, using the example of a retaliation script and a feeling accepted script, of the intended transformation of the hunting network into a positive association network**. Exposing to and contextualizing violent events is supposed to reduce the strength of associations between positive emotions and aggressive cues. Dotted lines indicate dissolving associations; solid lines represent persisting or reinforced associations. The figure has been developed based on the schematic representation of the interaction between fear network and hunting network that triggers aggressive behavior [from Crombach and Elbert (82)].

Narrative exposure therapy for forensic offender rehabilitation [FORNET; (77, 78)] not only aims to reduce PTSD symptoms but also to address aggressive behaviors by recalling the experiences through narrative exposure. Although offenders are not necessarily traumatized, many offenders have experienced traumatic events in their past. FORNET broadly follows the logic of the evidence-based trauma-focused NET (79–81). It helps the client to anchor not only fearful and traumatic experiences but also positive feelings that might have been linked to various forms of aggressive behavior in the past. FORNET aims also to deconstruct the hunting network by associating the positive emotions related to violent behavior with a specific context and time period. After working chronologically through the client’s past up until the present, the last session focuses on dealing with the past and with aggressive behavior. For former members of violent groups, e.g., militia groups, armies, or gangs, FORNET ends with a group session focusing on the role change from a violent offender to a citizen, who is capable of living a socially adjusted life. Additionally, visions for the future are developed to foster successful integration into society. Alternatively, an individual session completes the therapy focusing on reinforcing an associative network of positive emotions linking appetitive emotions to socially acceptable activities. Figure 2 represents schematically the process of transforming the hunting network into a non-violent positive association network.

Several studies have shown that NET can be effective within four to six sessions of 90–120 min (79–81). The effectiveness as a short-term intervention is essential for implementing NET and FORNET in unstable and resource-poor environments like a refugee camp or a region of ongoing conflict (83). FORNET can be successfully completed in six sessions (not including diagnostics and psycho-education). One session lasts on average 90 min.

### FORNET: Step by step

#### Lifeline

After psycho-education the therapy begins with the “lifeline” exercise. Following the logic of NET (76), the client lays out his path of life along a rope or string, which symbolizes the person’s life up until now. One end of the string stays coiled up and symbolizes the future. The client places flowers on the string for major happy events and good times in life and stones for fearful and traumatic events. In addition, we introduced sticks to symbolize active involvement in violent acts. In this way, combat, fight, and other such events were not colored by *a priori* moral judgment. Using the stick as a symbol also avoids imposing any particular emotional valence on the violent acts. This is important, as these are frequently emotionally ambivalent situations. For the violent acts, in particular, the therapist focuses on the first time they perpetrated violence (e.g., first fight, first killing, first rape). Additionally, the therapist asks about violent acts involving strong emotions, which are therefore easily cued by reminders (e.g., fight in which he felt most powerful, he felt most fear). If applicable, the entry into and the exit out of an armed group or gang both mark important moments in the life of the client. Thus, the entry and the exit of a violent group should be marked or at least mentioned during the lifeline exercise.

**Figure 3:**
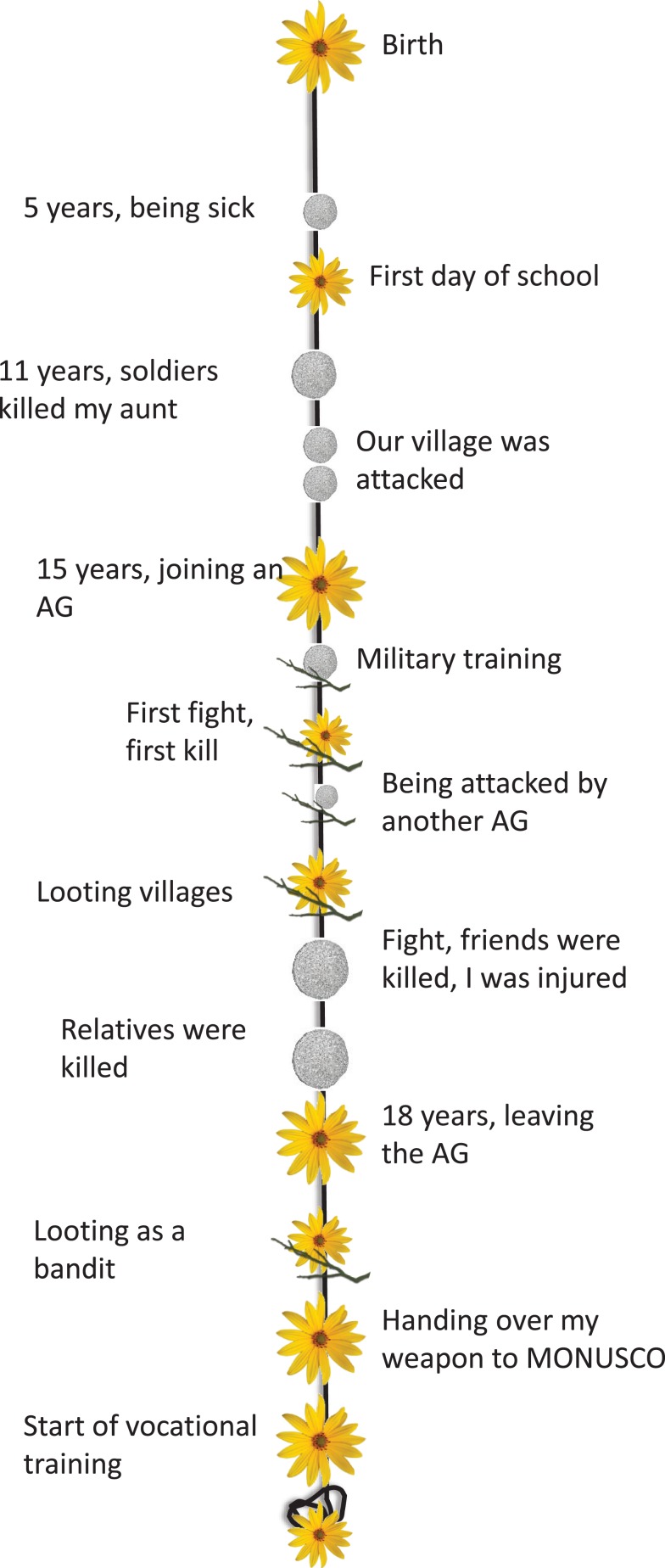
**Lifeline of a former child soldier (20 years) from the DR Congo**. AG, armed group; MONUSCO, United Nations Mission in the DRC.

The client is free to choose symbols and also to combine them. Hence, sticks can also be combined with stones or flowers to emphasize the complex emotions felt during the active involvement in violent acts. It is, however, important that they symbolize the emotions at the time of the event and not at the time of the therapy. The therapist encourages the client to give each symbol a heading or name and to clearly determine time and space of the event. It is important to place the symbols as much as possible in chronological order along the line. During the whole lifeline exercise, the therapist helps the client to stay on the cold memory side. The therapist needs to focus on facts and dates rather than on emotions and bodily sensations. The in-depth recall of every event will follow later on. The lifeline exercise serves only as an overview of important life events and as an orientation for the following sessions. If the client is very emotional while placing one symbol, the therapist acknowledges the client’s feelings and explains to the client that in the next sessions there will be enough time to deeply focus on this event. Then, the therapist should summarize the cold facts of this event and help the client to continue with identifying the next event. The lifeline exercise should be completed in one session. Figure 3 displays a lifeline of a former child soldier from the DRC.

#### Starting the Narration

The following sessions are closely based on the approach of NET. The therapist supports the client in following his lifeline chronologically from his birth to the present time. When the client is approaching a traumatic or violent event, the narration slows down and the therapist will begin to guide the client through the event in fine detail. Before talking about the event in detail, the context of the event must be clarified. Not only time and place are important but also the point that marks the beginning of the event. The in-depth recall of the event starts with the client imagining and narrating the beginning of the event. From this point, the event is recalled in very detail and slow motion. The narrative exposure of an event should never be interrupted and must always be completed in one session.

As in NET, the therapist has an active role and helps the client to relive the emotions, cognitions, and bodily sensations experienced during his most traumatic events (*stones*) and while perpetrating violent acts (*sticks*). These features of the hot memory are connected to the autobiographic memory by putting all of the memory fragments into words. The therapist also guides the client to contrast between now and then. Besides the feelings and cognitions of the past, the client’s current view of the event, including his thoughts, feelings, and bodily sensations, is taken into account. The memorized feelings (e.g., *then I was shocked and afraid*) are contrasted with the feelings that arise in the here and now when the memories are recalled (e.g., *when I think back, I get angry*). In this way, the therapist helps the client to anchor the cognitions and emotions that are recalled with the event in the past. Retelling an experience in such a way leads to a new and enhanced memory consolidation and ultimately causes associative networks to dissolve as both, traumatic experiences and perpetrated violent acts, become integrated into the memory.

##### Example: extract of a narration of a stick

Using the infrared camera I could see someone lying in a bush. I shot several grenades in this direction. Five to six people were standing behind the antenna installation. I shot a grenade in their direction. Directly after I volleyed several grenades. Via radio I could hear that the special unit complained about the mortar fire. The last one I could see tried to circle our car at a distance of 600 meters. He was wearing brownish typical Afghan clothing. I shot a grenade in his direction. Just before the grenade banged into him, I could see the perplexed face of this motherfucker. I was very happy and laughing. On the screen I could check that there was no one still moving. In the car there was an upbeat atmosphere and we felt in a superior position. It took a while to come back to reality. [.]. The situation was calm again and I drank a coke (Soldier, after deployment in Afghanistan).

##### Example: punishment of a thief

In the beginning I was very angry and I wanted to punish him (the thief), but then I started to enjoy beating him. I felt the need to harm him. I took a hammer and a dull nail and started to torture him. I have beaten the nail again and again in his feet. I do not even know how often I did this. Every time he was screaming. I continued all night to beat him with a stick and to torture him with the hammer and the nail. He was bleeding everywhere and cried that we would kill him. I felt joy everywhere in my body and was very satisfied. I was pleased to hurt him. I felt powerful. The feeling was like winning a football game against a very strong team. I did not want to stop […] [juvenile offender, 21 years old, from Burundi; (58)].

The continuous process of re-experiencing the emotions, cognitions, and bodily sensations, while putting everything into words and into a coherent narration, will lead to reduced arousal when being confronted with triggers. Emotional impact and bodily arousal will decrease over time. The therapist should never end a session before some reduction of arousal has taken place. The highest level of emotional arousal must have been reached and a considerable reduction of fear and excitement is necessary before a session may be ended. However, a full reduction of arousal cannot be expected after one session. Further reduction of fear and excitement takes place in the following sessions, when the events are reviewed again. A reduction of emotional and bodily arousal will also take place between sessions because the client will continue to think about the events.

The therapist transcribes the narrative using the past tense and the client’s own expressions. In the beginning of the following sessions, the therapist reads the narrative to the client who is asked to relive the event again and to correct and add details. If the client lives in an insecure setting or instable security situation, FORNET can also be performed without a written narrative. In this case, an oral narration replaces the reading of the written narrative in the beginning of the following sessions. In each case, it is crucial to follow the chronological order of the events. The client might tend to make judgments about the emotions that arise. The therapist encourages the client to perceive the emotions during exposition without judgment, and acts as a role model for this. The unconditional acceptance of every emotion by the therapist is essential for the exposure of violent events. Both the recall of positive and negative affective responses is encouraged even when the worst offenses are recalled. The therapist encourages the client to verbalize and relive all of the feelings connected to perpetrated violent acts. It is absolutely essential that the therapist adopts an accepting and supporting rather than judgmental position. After the exposition of a violent act, an attribution of meaning from the client’s current point of view can be elaborated. The unconditional acceptance of every emotion can be very challenging for therapists while working with violent offenders. This requires a solid therapeutic training and regular peer-supervision and external supervision. Not every therapist feels confident and able to work with violent offenders. FORNET should only be started when the therapist is confident that this therapeutic approach and the emotional challenges it poses is compatible with the therapist’s values, ethics, and competence.

#### Choosing Events

Given the limited number of sessions, it is often essential to select, the events that are most important to the client. The lifeline exercise can assist the therapist and client in making these decisions. The therapist focuses on the most traumatic events and specific perpetrated violent acts that are connected to strong emotions and positive (sensation of being powerful) or negative arousal (fear). Additionally, the first time the client acted aggressively and committed a certain type of offense are often important events and should therefore be selected. For example, the first time the client attacked someone marks an important turning point in his life in which he may have overcome the learned inhibition to kill or severely injure another human. Therefore, it is important to go through the first attack or killing in great detail, to emphasize subsequent changes in the case of repeated violent acts. Again, the therapist supports the client to recall the event in fine detail and in chronological order. The therapist fully explores all emotions, both negative and positive, which are linked to the specific event (e.g., primary emotions: disgust, fear, or joy; self-conscious emotions: guilt or pride). During the first attack or killing, it often happens that the client becomes keenly aware of his own vulnerability. This cognition should be verbalized during therapy along with any sensations, including the description of the victim (*What did the victim look like? Did he scream? Did he bleed?*).

##### Example: first killing

We were hidden between trees. We were only two, my friend and I. We lay down. It was dark. I felt the wet grass under me. Suddenly a soldier approached us. He did not see us. It was an enemy. He was a grown-up soldier. I was a bit afraid. My heart was bumping a bit faster. We watched him as he came closer. I thought: “This must be a spy of the enemy. We have to kill him.” I took my knife and targeted at the enemy. I was excited. It felt like a game, but I also felt a bit of fear. I threw the knife and hit the enemy in the neck. It was a big knife and I really hit him. I was feeling proud. The enemy fell down. I felt happy that I was able to hit him. […] My friend jumped out of the bush, took the spy’s gun and shot him. […] Then we rushed back to the camp. We were afraid to be punished because we were supposed to capture but not to kill the spy of the enemy. When we arrived we were praised and I felt very powerful and proud. [child soldier, 12 years, from the DR Congo; (78)]

Finally, the therapist and client focus on how the client overcame the inhibition threshold to kill or injure another person. The therapist concentrates on cognitions (e.g., outgroup, enemy) and emotions (e.g., fear, anger, feelings of hatred, or revenge) that made the client overcome this threshold. Subsequently, the client is encouraged to mention his current thoughts, feelings about the event, and the meaning for him and his life. However, in some cases, the meaning of the event may not have changed and it may still activate positive emotions. If this is the case, the therapist should continue being non-judgmental. In FORNET, the integration of the experiences into the autobiographical memory is most important.

#### Ending the Narration

During the last exposure session, the autobiography finally reaches the present and the narration of the most emotionally arousing events in chronological order is thus completed. With the help of the therapist, it is now possible for the client to understand his development across his whole life. This provides a strong basis for discussing future developments. At the end of the therapy, the therapist and the client also elaborate hopes and wishes for the future.

The written narrative is read one last time. A final corrected version of the written narrative is signed by the therapist and the client and handed over to the client. In case FORNET was performed without a written narrative, the lifeline exercise can be repeated as a closing ritual. Flowers representing wishes for the future can be placed on the end of the rope that remains curled up, representing the future.

#### Final Session

Forensic offender rehabilitation is completed by one or more sessions that focus on the client’s current situation, supports rehabilitation and prevents further violent offenses. Depending on the client’s needs and life, a group or an individual setting may be chosen. Dismantling studies would be needed to see the relevance of this NET-supplementing and future-oriented treatment module. However, in order to prevent relapse of criminal offenses, it seems plausible that emphasis on this element should be put in future studies whenever sufficient resources are available.

##### Group sessions for rehabilitation of former members of violent groups

The group session focuses on the challenges that go along with a change of role and identity. Groups, such as gangs, armies, or other armed groups, often present a very important part of the identity of the members. When they cease to be a part of such a group, former members need to adjust to that change and orient to their new role. In the group session, this role change is addressed and reinforced. A group consists of three to four clients and one therapist. The therapist structures and guides the discussion, encouraging the clients to hold and discuss different views and to be open to the experiences of others. Furthermore, the therapist encourages them to take responsibility for their own lives and to develop aims to foster successful rehabilitation.

At first, the clients review their own lives within the former group and discuss the positive and negative aspects of being part of this group. At this point, the old role as a group member is discussed in a broader sense, as clients might not wish to disclose specific experiences, which were addressed during the individual sessions. Subsequently, the therapists focus on the role change and on the connected feelings and emotions of the clients (e.g., *How difficult was it to hand over your weapon? How did you feel when you actually did it? How do you feel about it right now?*). In the following part, the therapists direct the discussion to the current situation. The clients discuss positive and difficult aspects of their current life and identify advantages of the current life in comparison to being a former group member. The therapists encourage them to develop strategies together to overcome their difficulties. The group session ends with future plans and wishes of each client and thoughts on the realization of these plans.

##### Examples of questions structuring the group session

- What was positive in the old role (e.g., as a soldier)? How did you feel? Was it the same for the others?
- What was negative in the old role (e.g., as a soldier)? How did you feel? Was it the same for the others?
- What changed when you left your group? (e.g., Was it difficult to hand over the weapon?) How did you feel? Was it the same for the others?
- What is good/better in the new role (e.g., as a civilian)? Is it the same for the others?
- What are the difficulties in the new role (e.g., as a civilian)? What can you yourself do about it? How can you help each other?
- What are your plans for the future? How can you get there?

##### Individual ending session: reinforcing a positive associative network

As an alternative or an addition to the group format, the individual ending session aims to develop perspectives for the future and to reinforce an associative network of positive emotions with socially acceptable activities. This approach aims to strengthen the client’s self-esteem and sense of self-efficacy (82). The therapist assists the client to structure his/her perspective of the future by guiding him/her with the following questions: (a) *What aims and wishes do you have for your future?* (b) *What difficulties and obstacles do you see?* and (c) *What are your personal strengths that will help you to overcome the obstacles and achieve your goals?* During the last session, the client experiences that his/her plans and hopes matter and that there is a person who cares about his/her future, which helps the client to develop a more positive outlook.

Additionally, the therapist encourages the client to recall a recent exciting moment that was associated with feelings of power, enjoyment, pride, and control – emotions that may have been experienced previously during aggressive acts. The therapist helps the client to identify a socially appropriate situation (e.g., being welcomed by family or peers, scoring a goal during a soccer game or succeeding in a school exam). The therapist guides the client to experience the associated positive emotions as strongly as possible. When the client is most enthusiastic, feeling exhilarated, strong, and powerful – during the positive equivalent of the “hot spot” – the therapist stops the narration. While the client still experiences these positive emotions, the therapist summarizes his/her personal strengths and provides positive feedback aiming to provide support and increase his/her self-esteem. The emotionally aroused state of the participant at the end of the session may facilitate the integration of environmental cues of past and present in an associative network representation. Ending the narration during the most exciting moment in a socially appropriate situation aims to integrate positive feelings that might previously have been part of the hunting/appetitive network into a non-violent positive association network (see Figure 2). In consequence, these positive emotions may be more easily triggered by socially accepted cues because a generalization in non-violent surroundings may take place. Hence, client might be able to access the positive emotions easier.

### Feasibility, effectiveness, and the potential to disseminate

In a first randomized controlled clinical trial, with the aim of reducing traumatic stress and appetitive aggression 15 ex-combatants received FORNET and were compared to a matched control group who received “treatment as usual” in a reintegration center of war-affected youth in the eastern DRC. The treatment group reported reduced PTSD symptoms and less contact with active and former combatants 6 months after the treatment. Appetitive aggression decreased substantially in both groups (78). In a randomized controlled clinical trial with violent youth at a Burundian residential center for former street children, 16 youths who received FORNET reported having committed significantly fewer offenses and presented with fewer physical-health complaints than did their matched control participants 4–7 months after treatment (82). These pilot studies proved the feasibility of FORNET, found first evidence of a positive outcome, and highlighted the importance of addressing the whole range of experiences while treating former combatants or juvenile offenders.

In a very recent semi-randomized trial in the eastern DRC, FORNET was conducted by local counselors trained by experts (phase 1) and by experienced counselors (phase 2). In total, 98 demobilizing combatants were treated using FORNET; treatment-as-usual served as the control condition. Six months post-intervention, FORNET significantly reduced PTSD symptoms. Beneficial effects were also found for depression severity and drug dependence. Effects for reintegration indices were moderate to small. All treatment gains were retained at 12 months. Thus, individuals without previous training in psychotherapy (but with expertise related to combat and armed groups or gangs) can learn to effectively apply FORNET and support the rehabilitation process of soldiers and violent offenders (Köbach et al., under review).

### Perspectives and possible applications of FORNET

Currently, our team is completing studies in South Africa and Burundi to further test the effectiveness and the practical relevance of FORNET. In South Africa, a randomized controlled trial is investigating the effectiveness of FORNET in a sample of adolescent criminal offenders in the townships of Cape Town, which is conducted under conditions where clients remain continuously exposed to ongoing stressors, including the continuous threat of traumatic stressors. As dissemination is key to community-based care, a train-the-trainers model is also being evaluated to establish whether the psychotherapeutic techniques have been successfully implemented.

In order to assess gender effects as well as implications of attachment issues and childhood maltreatment in greater detail, we have been offering FORNET to male and female ex-combatants in Burundi. In this study, we also aim to investigate how contextualizing appetitive aggressive cues and reconnecting rewarding emotions with socially acceptable activities may interact to diminish involvement in violent behavior. The results of these studies will further deepen our understanding of mechanisms that contribute to a cycle of violence and help implement specific interventions to interrupt potential trans-generational effects.

Over the past 50 years, research has shown that experiencing violence particularly during childhood is strongly associated with subsequent delinquency and aggressive behavior (36, 39). Hence, treating trauma-related mental health issues in violent offenders seems to be crucial. At the same time, recent studies have shown that dealing with rewarding emotions that are related to aggressive behavior is equally important (58, 59). Most common risk factors, such as childhood maltreatment, exposure to violence, and committing aggressive acts, are, however, not only limited to populations in war and conflict settings, such as demobilized combatants or children and adolescents living in the streets of post-conflict countries but they also apply to soldiers, veterans, and violent offenders all over the world. The existing evidence for FORNET suggests that addressing violent behavior by means of NET is a promising approach to treat traumatized soldiers, veterans, and violent offenders and to support them to regulate their desire for aggressively acting out. Hence, FORNET might be a beneficial addition to treatment programs for soldiers or veterans after war deployment. Furthermore, it may be implemented in forensic settings, such as forensic psychiatry and prisons, fostering the rehabilitation of violent offenders.

### Ethical statement

All persons that we have quoted in the section on FORNET have received treatment. The child soldiers and the juvenile offender were treated in one of our randomized controlled clinical trials. They signed an informed consent and the intervention procedure was approved by the local authorities and the Institutional Review Board of the University of Konstanz, Germany. Moreover, juvenile offender and the soldier gave their informed and explicit consent that we are allowed to publish these quotes. Finally, the chair of the Institutional Review Board of the University of Konstanz approved the publication of the quotes in the presented form.

## Conflict of Interest Statement

The authors declare that the research was conducted in the absence of any commercial or financial relationships that could be construed as a potential conflict of interest.
